# The impact of alcohol among injury patients in Moshi, Tanzania: a nested case-crossover study

**DOI:** 10.1186/s12889-018-5144-z

**Published:** 2018-02-21

**Authors:** Catherine A. Staton, Joao Ricardo Nickenig Vissoci, Nicole Toomey, Jihad Abdelgadir, Patricia Chou, Michael Haglund, Blandina T. Mmbaga, Mark Mvungi, Monica Swahn

**Affiliations:** 1 Division of Emergency Medicine, Duke University Medical Center, Box 3096, 2301 Erwin Road, Durham, North Carolina USA; 2 Duke Global Health Institute, Duke University, Box 3096, 2301 Erwin Road, Durham, North Carolina USA; 3 National Institute of Alcohol Abuse and Alcoholism, Washington, DC, USA; 4 Duke Global Neurosurgery and Neurology Division, Department of Neurosurgery, Duke University, Durham, North Carolina USA; 5 Kilimanjaro Christian Medical Center, Moshi, Tanzania; 6 Division of Epidemiology and Biostatistics, School of Public Health, Georgia State University, Atlanta, Georgia USA

**Keywords:** Alcohol use, Injury, Tanzania

## Abstract

**Background:**

Globally, alcohol is responsible for 3.3 million deaths annually and contributes to 5.9% of the overall global burden of disease. In Sub-Saharan Africa, alcohol is the leading avoidable risk factor accounting for a substantial portion of death and disability. This project aimed to determine the proportion of injuries related to alcohol and the increased risk of injury due to alcohol among injury patients seeking care at the emergency department (ED) of Kilimanjaro Christian Medical Centre (KCMC) in Moshi, Tanzania.

**Methods:**

A representative cross-sectional sample of adult patients presenting to the KCMC ED with acute injury were enrolled in this study with a nested case-crossover design. Patient demographics, injury characteristics, and severity as well as alcohol use behaviors were collected. Alcohol breathalyzers were administered to the enrolled patients. Data on activities and alcohol use were collected for the time period 6 h prior to injury and two control periods: 24–30 h prior to injury and 1 week prior to injury.

**Results:**

During 47 weeks of data collection, 24,070 patients were screened, of which 2164 suffered injuries, and 516 met the inclusion and exclusion criteria, consented to participate, and had complete data. Of the study participants, 76% were male, and 30% tested positive for alcohol on arrival to the ED. Alcohol use was associated with being male and being employed. Alcohol use was associated with an increased risk of injury (OR 5.71; 95% CI 3.84–8.50), and specifically road traffic injuries were associated with the highest odds of injury with alcohol use (OR 6.53, 95% CI 3.98–10.71). For all injuries and road traffic injuries specifically, we found an increase in the odds of injury with an incremental increase in the dose of alcohol.

**Conclusions:**

At KCMC in Moshi, Tanzania, 3 of 10 injury patients tested positive for alcohol on presentation for care. Similarly, alcohol use conveys an increased risk for injury in this setting. Evidence-based prevention strategies for alcohol-related injuries need to be implemented to reduce alcohol misuse and alcohol-related injuries.

## Highlights

- 30% of patients seeking acute injury care in Tanzania tested positive for alcohol.
- Alcohol use is associated with a 5-fold increase in the odds of injury (OR 5.71).
- Alcohol appears to have a dose-dependent increase in the odds of injury in Tanzania.

## Background

Globally, alcohol misuse is responsible for 3.3 million deaths annually and contributes to 5.9% of the overall global burden of disease and injury. Approximately one-third of all alcohol-attributable disability-adjusted life years lost are due to injuries and 45% of alcohol-attributable deaths are due to violence [1, 2]. Additionally, in 2004, the global burden of injury and disease attributable to alcohol was 7.6% for men and 1.4% for women [3]. In Sub-Saharan Africa, alcohol misuse is a leading avoidable risk factor accounting for a substantial portion of the region’s global burden of death and disability [1, 4, 5]. Drinking behaviors and patterns in the African region are the second worst worldwide due to high rates of binge drinking and alcohol dependence [1, 4, 5]. For the same pattern of drinking, individuals from a lower socioeconomic setting have a higher alcohol attributable mortality and burden of disease and injury [2]. Excessive alcohol use has been associated with many high-risk behaviors, such as crime, aggressive driving, interpersonal violence, unintentional injuries, and self-inflicted injury [6]. Similarly, violence and injury victims are more likely to use alcohol to cope with the experience of victimization or injury [7, 8].

Few surveillance systems, if any, routinely collect data among injured patients in emergency departments (ED) in Sub-Saharan Africa. Accordingly, little is known about the proportion of patients who have consumed alcohol and the extent to which alcohol increases injury risk. According to the literature and for our study, an alcohol-related injury is typically defined as an injury with a positive breathalyzer test (BAC (≥.01%) for alcohol within 6 h of the injury. [9, 10] We adopted this strict alcohol level because of extensive delays common in care seeking in low- and middle-income countries as reported in the literature [11], which cites an increase in injury, especially road traffic injury (RTI), with any amount of alcohol use. Across studies, the proportion of patients presenting to an ED for treatment of their injury who suffer from alcohol-related injuries ranges from 6%–45%, depending on the country [9, 12]. These patients who have experienced harmful health consequences, or ‘harmful alcohol users,’ are at a high risk of repeated alcohol-related harm and could benefit from further harm reduction interventions. In addition, prior pooled global studies have shown that a positive BAC (≥.01%) conveyed a 5.7-increase in the odds of injury (95% CI: 4.5–7.3), but only one of these studies was from the unique Sub-Saharan African context and none was from Tanzania [9, 13].

Moshi, Tanzania, a popular tourist destination at the base of Mount Kilimanjaro, has increasing alcohol consumption rates, minimal government oversight, and rising injury rates mainly fueled by road traffic injuries from motorcycle crashes [14–16]. Tanzanian national statistics cite an 81% abstinence rate from alcohol, compared with the World Health Organization’s report of a 58% abstinence rate in the region. [2] While it appears that the majority of the population abstains from alcohol, likely due to religious reasons, the WHO reports an annual average consumption of 7.7 L per capita of pure alcohol, with 34% of drinkers (41% male, 23% female) partaking in heavy episodic drinking. [2] These statistics suggest that a high proportion of drinkers partake in high-risk drinking behaviors that are highly linked to alcohol related injury or other harm. In our previous preliminary research at KCMC, approximately 30% of injury patients had alcohol-related injuries per clinical evaluation or self-report [15].

Given the high burden of injury and the high rates of high-risk alcohol use, this study aimed to determine the proportion of injured patients at the KCMC ED with a positive breathalyzer through a representative cross-sectional sampling of injury patients and assess the increased risk of injury due to alcohol through a nested case-crossover control method.

## Methods

### Study design

This study was organized in two phases: (1) a representative cross-sectional sample of injury patients presenting to the KCMC ED to inform the proportion of alcohol-related injuries and injuries characteristic; and (2) a nested case-crossover design to determine the risk of injury due to alcohol. This study’s case-crossover methods were based on the WHO Collaborative Study on Alcohol and Injuries protocol, which has been described in detail elsewhere [17–19]. In brief, self-reported alcohol use in the 6 h prior to injury was compared with alcohol use during two control time periods, 24–30 h and 1 week prior to injury, to determine the increased risk of injury due to alcohol.

### Study sample

We enrolled patients seeking care at the KCMC ED, located in Moshi, Tanzania, in the Kilimanjaro Region in Northern Tanzania. KCMC is a referral and teaching hospital for the region, serving over 15 million people. To be included in the study, patients must have been ≥18 years of age, sought care for an acute injury at the KCMC ED within 6 h of the injury, determined to be clinically sober by the treating physician or had a proxy consent, and been able to converse with the research personnel or had a proxy who was willing to answer questions on behalf of the injured patient. If a patient was too ill or intoxicated on arrival, but had a Legal Authorized Representative (LAR) available to administer informed consent, the patient was included in the study. Subjects who were deemed unable to give consent by their treating physician due to injury or intoxication were enrolled by LAR and were re-consented when they regained capacity to consent. Exclusion criteria were a patient or family refusing to respond to any of the assessments; patients who were too ill, in too much pain, or without an LAR to be enrolled in the study; and patients who presented outside of the 6-h time window or were less than 18 years of age. Ethics approval was obtained by the Kilimanjaro Christian Medical University College Ethics Review Board, the Tanzanian National Institute of Medical Research and the Duke University Institutional Review Board. All participants in this project provided informed consent according to our ethics governing bodies requirements.

### Data collection

All data were collected during a one-time interview of patients presenting to KCMC for treatment of acute injuries. Patients were approached once they were medically stable. During rotating enrollment shifts (morning, evening, and night), all patients presenting for care were approached to obtain a representative sample of patients presenting to the ED. If patients met the inclusion and exclusion criteria, they were offered enrollment in the study. After providing informed consent personally or by LAR, patients were administered a breathalyzer test. Administration of the surveys in the local language of Kiswahili either followed immediately after the breathalyzer or for some patients enrolled by LAR at a later time when he/she was deemed clinically sober and able to consent and participate. Study data were collected and managed using REDCap electronic data capture tools hosted at Duke University. [20].

### Sample size calculation

This study included a basic cross-sectional study design and a nested case-crossover design. The sample size required for the cross-sectional study to assess positive alcohol breathalyzers or those individuals with alcohol-related injuries was estimated to be *N* = 323 injury patients. This sample size would allow us to estimate our anticipated 30% proportion based on our prior work to within +/− 5% (95% confidence interval) [15, 21]. For a nested case-crossover design, we estimated that we needed to enroll 140 cases, each of whom would serve as their own controls in a 2 control time periods to 1 case proportion. We assumed a 30% proportion of alcohol-related injuries, based on our previous work. [21] With this sample size, we estimated a 90% power to detect an odds ratio (OR) of 2.0 [18], with 0.05 significance. To ensure further analysis of subgroups, we chose to over enroll study participants.

### Variables

We collected patient demographic characteristics (age, gender, education, and employment) and injury characteristics, including time since injury, injury severity, type of injury, and mechanism of injury, including type of RTI and location of injury. Injury severity was determined by the Kampala Trauma Score (KTS) and the Revised Trauma Score (RTS). Both scales have been validated to predict outcomes in both high-income and Tanzanian injury settings. [15, 21] Both scales were used because RTS is not commonly used clinically, and the KTS is not universally used limiting the ability to compare our data internationally. The alcohol-related data we collected were breathalyzer testing where BAC (≥.01%) is positive for alcohol at the time of presentation to the hospital for care post-injury, self-reported alcohol use during the three specific time periods (within 6 h of injury, 24–30 h before injury, and 1 week before injury), and dosage of alcohol by container following the WHO guidelines for containers [18]. Patients were asked, *“In the 6 hours leading up to your injury, did you have any alcohol, even one drink? (yes/no)”.* For the control periods, patients were asked*, “Think about what you were doing yesterday at the exact time of your injury; did you have any alcohol to drink in the 6 hours leading up to this time? (yes/no)”* and *“Think about what you were doing 1 week ago at the exact time of your injury; did you have any alcohol to drink in the 6 hours leading up to this time? (yes/no)”.* Self-reported alcohol use data were used for the nested case crossover analysis. Additionally, activities and alcohol use were self-reported for the three time periods of interest.

### Cross-sectional data analysis

Data were missing for 51 (10%) cases, in different variables collected. However, the amount of missing data across variables ranged from 0.1% to no more than 5%. Missing data were imputed using multiple imputation [22]. We used descriptive statistics to calculate the frequencies of demographic, injury, and alcohol data of all injury patients. Comparisons between the alcohol-positive and alcohol-negative injury patients were performed using Chi-squared or Fisher’s exact testing for the categorical data and t-test for the numeric data. Multivariate logistic regression was used to verify the association between demographic and injury data and alcohol use. Initially, all demographic and injury variables were included in the multivariate model. A sensitivity analysis was conducted by backward elimination for the variables with non-significant bivariate association or indicators of collinearity. We reported the full model with all indicators because the changes in the sensitivity analysis did not significantly improve the model by comparing the distribution of the residuals and the Akaike’s Information Criterion values. KTS and RTS indicated collinearity, as expected, but both variables were not significantly associated with alcohol use in the bivariate analysis, and the sensitivity analysis did not improve the model by eliminating the variables from the model.

### Nested case-crossover analysis

Similar to prior WHO Alcohol and Injury Collaboration analyses, [17, 18] we compared injured patients’ self-reported alcohol use in the 6 h prior to injury with alcohol use during two control time periods (24–30 h prior to injury and 1 week prior to injury), following a self-controlled matched-pair case-crossover design [18, 19, 23]. Because injured patients are their own controls, this study design controls for characteristics that may affect the risk of an injury but do not change over a short time period [24]. For each case of injury, conditional logistic regression models with 1:1 and 1:2 self-controlled matched-pair case-crossover designs were used; the statistical analysis compared the case time period (6 h prior to injury) versus two potential control periods (24–30 h prior to injury and 1 week prior to injury. Both control periods in a 1:2 comparison were used because while 1-week measurements may account for weekly drinking patterns, it has more potential recall bias than a control time period of 24–30 h. [25, 26] All data were calculated and reported in ORs, 95% confidence intervals (CIs), and *P* values with 5% significance. A subgroup analysis was performed for RTI and violence. The data analysis was performed using R in the survival package [27].

## Results

**Fig. 1:**
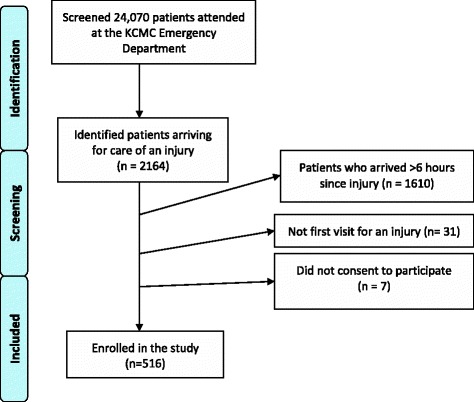
Flowchart of Enrollment

During 47 weeks of data collection from August 5, 2013 to July 21, 2014, 24,070 patients were screened, of which 2164 suffered injuries. Of these patients, 1610 (74.4%) did not arrive within 6 h of injury, 31 patients (1.4%) presented for a repeat or follow-up visit, and 7 (0.3%) patients did not consent to participate. Ultimately, 516 patients met all the inclusion and exclusion criteria and consented to participate, as seen in Fig. 1.

### Predictors of alcohol use prior to injury

As seen in Table 1, the study sample was composed mainly of males (76.4%) with a mean age of 34.4 (SD 13.3) years, working registered jobs (80.8%), and with a mean 9.7 (SD 9.5) years of education. Among participants, 30.0% (95% CI, 26.3–34.4) had a positive breathalyzer at the time of injury. Overall, 15.1% of the injured patients and 48.1% of the BAC positive patients were above the Tanzanian legal alcohol limit of 80 mg/dL per breathalyzer testing. The proportion of patients who self-reported alcohol at 24–30 h and 1 week prior to injury were 16.0% and 12.0%, respectively. Two participants who were BAC positive reported not drinking in the last year prior to injury. Participants reporting alcohol use were more likely to be male and employed (Table 1). Participants who reported alcohol use during the control time periods were more likely to have used alcohol at the time of the injury.

**Table 1:** Injury characteristics and association with positive alcohol use prior to arriving at the ED

	Total (*N* = 516)	BAC Positive (*N* = 156)	BAC Negative (*N* = 360)	P Value	AOR	CI 95%	P value
*Sociodemographics*							
Age (years), Mean (SD)	34.4 (13.3)	35.2 (11.2)	33.9 (14.1)	0.31	1.01	(1.00–1.03)	0.152
Male, N (%)	394 (76.4)	135 (86.5)	259 (72.0)	*< 0.001* ^*^	*2.29*	*(1.31–4.15)*	*0.004*
Education (years), Mean (SD)	9.6 (9.5)	10.3 (10.1)	8.7 (7.9)	0.123	0.98	(0.94–1.01)	0.271
Employed, N (%)	416 (80.6)	141 (90.4)	274 (76.4)	*< 0.001* ^*^	*0.46*	*(0.24–0.85)*	*0.016*
*Injury characteristics*							
Time since injury (hours), Mean (SD)	2.6 (1.4)	2.8 (1.5)	2.5 (1.4)	*0.034* ^*^	1.09	(0.94–1.27)	0.244
KTS, Mean (SD)	14.3 (0.9)	14.2 (0.9)	14.3 (0.9)	0.395	1.03	(0.74–1.44)	0.841
RTS, Mean (SD)	7.7 (0.5)	7.7 (0.4)	7.7 (0.6)	0.831	1.12	(0.63–2.23)	0.719
Unintentional Injury	440 (85.3)	122 (78.2)	318 (88.3)	*0.003* ^*^	3.33	(0.91–14.0)	0.081
Type of injury							
Fracture	185 (35.8)	61 (39.1)	124 (34.4)	0.311	1.45	(0.89–2.37)	0.138
Dislocation	80 (15.5)	16 (10.3)	64 (17.7)	*0.050* ^*^	0.8	(0.40–1.52)	0.501
Open Wound	197 (38.2)	79 (50.6)	118 (32.8)	*0.030* ^*^	*2.04*	(1.29–3.26)	*0.003*
Bruise	111 (22.5)	28 (17.9)	83 (23.1)	0.195	0.74	(0.43–1.27)	0.282
Concussion	149 (28.9)	46 (29.5)	103 (28.6)	0.84	1.28	(0.77–2.10)	0.341
Organ Injury	56 (10.9)	14 (9.0)	42 (11.7)	0.366	0.94	(0.43–1.96)	0.87
Mechanism of Injury							
RTI	375 (72.7)	109 (69.9)	266 (73.9)	0.245	Ref		
Violence	88 (17.0)	33 (21.1)	55 (15.3)		0.26	(0.05–1.10)	0.08
Fall/Trip	53 (10.3)	14 (9.0)	39 (10.8)		0.67	(0.25–1.69)	0.403
Location of Injury							
Outdoor public place	399 (77.3)	118 (75.6)	281 (78.1)	*< 0.001*	Ref		
Drinking place	17 (3.3)	13 (8.3)	4 (1.1)		*9.27*	*(2.38–44.9)*	*0.003*
Home	45 (8.7)	17 (10.9)	28 (7.8)		1.88	(0.72–4.97)	0.2
Work place	41 (8.0)	4 (2.6)	37 (10.3)		*0.26*	*(0.07–0.81)*	*0.031*
Other	14 (2.7)	4 (2.6)	10 (2.8)		1.18	(0.29–4.17)	0.802

*p*-value is a comparison between BAC positive and BAC negative

*VRU* Vulnerable road user, *RTI* Road traffic injury, *BAC* Breathlizer Alcohol Positive

*Significant at *P* < 0.05

Italics indicate *p* < 0.05

The injury severity was high overall and not significantly different based on BAC for KTS (14.3) and RTS (7.7) (Table 1). On average, patients arrived at the ED within 2.6 h (SD 1.4) with unintentional injuries (85.3%) mainly due to RTI (72.7%) and violence (17.0%), occurring in outdoor public spaces (77.3%). Fractures (35.8%) and open wounds (38.2%) were the most common injury types. A positive BAC test was significantly associated with higher proportions of open wounds and lower proportions of dislocations, and a higher proportion of intentional injuries. Multivariate regression models, controlling for age, education, and employment status, showed that patients with open wounds had approximately two times higher odds of having used alcohol prior to injury (OR 2.04; 95% CI 1.29–3.26) (Table 1). Patients injured at a drinking place (e.g. bar or restaurant) were more likely to have positive alcohol use at ED arrival (OR 9.27; 95% CI 2.38–44.90), whereas patients who suffered injuries at the workplace were less likely to have positive BAC at ED (OR 0.26; 95% CI 0.07–0.81) admission (Table 1) compared with injuries that occurred at open public spaces.

### Nested case-crossover analysis

As seen in Table 2, of those patients who consumed alcohol in the year prior to injury, 21.1% of the sample reported drinking more than 5 standard drinks at the time of injury, 13.6% drank over 5 drinks the day before injury, and 6.5% drank 5 drinks the week before the injury. In comparison, a higher proportion of BAC-positive injury patients reported having more than 5 drinks at the time of injury (36.6%), the day before injury (18.0%), and the week before the injury (8.3%). Overall, 23.7% (37) of the breathalyzer-positive participants reported not drinking 6 h prior to arrival.

**Table 2:** Self-reported alcohol consumption within 6 h of injury, 24–30 h prior to injury, and 1 week prior to injury for ‘past year drinker’ and alcohol-positive injury patients

	Alcohol use (# standard drinks^a^)	Within 6 h of injury, n (%)^b^	24–30 h before injury, n (%)^b^	1 week before injury, n (%)^b^
Past year drinkers (*n* = 279, 54.1% of total patients)	0	152 (54.5)	196 (70.3)	223 (79.9)
	1–2	25 (9.0)	22 (7.9)	15 (5.4)
	3–4	43 (15.4)	23 (8.2)	23 (8.2)
	5 or more	59 (21.1)	38 (13.6)	18 (6.5)
BAC positive (*n* = 156, 30% of total patients)	0	37 (23.7)	98 (62.8)	118 (75.6)
	1–2	20 (12.8)	12 (7.7)	9 (5.8)
	3–4	42 (26.9)	18 (11.5)	16 (10.3)
	5 or more	57 (36.6)	28 (18.0)	13 (8.3)

^**a**^A standard drink is 12 oz of 5% alcohol beer, 8 oz of 7% malt liquor, 5 oz of 12% alcohol wine or 1.5 oz of 40% alcohol liquor obtained by self-report

^b^Time periods include the 6-h window prior to injury, 24–30 h before injury, and 1 week before injury

The matched-pair analysis with a nested case-crossover design showed an increased odds of injury due to alcohol (OR 5.71, 95% CI 3.84–8.50). When stratifying by type of injury (Table 3), the odds of injury was 6.53 for RTIs (95% CI 3.98–10.71) and 5.31 for intentional violence-related injuries (95% CI, 2.24–12.57). Overall, any amount of alcohol use prior to injury was associated with the highest odds of injury. All patients and road traffic injury patients had a more apparent dose-dependent increase in the odds of injury compared to patients who suffered intentional violence.

**Table 3:** Alcohol use in the 6 h prior to injury according to type of injury and number of drinks

Number of standard drinks	All injury patients	Intentional violence	Road traffic injury only
	OR 95% CI	OR 95% CI	OR 95% CI
Any alcohol vs. no alcohol	5.71 (3.84–8.50)^**^	5.31 (2.24–12.57)^**^	6.53 (3.98–10.71)^**^
1–2 drinks^a^	2.22 (1.21–4.08)^*^	3.17 (0.75–13.4)	2.18 (1.06–4.50)^*^
3–4 drinks^a^	3.95 (2.24–6.96)^**^	4.00 (1.20–13.30)^*^	4.90 (2.44–9.84)^**^
5 or more drinks^a^	4.98 (2.88–8.60)^**^	3.90 (1.41–10.75)^*^	5.34 (2.57–11.10)^**^

A standard drink is 12 oz of 5% alcohol beer, 8 oz of 7% malt liquor, 5 oz of 12% alcohol wine or 1.5 oz of 40% alcohol liquor obtained by self-reporting

^**a**^Reference = 0 drinks

*P < 0.01

**P < 0.001

## Discussion

This is one of the first and largest studies to assess alcohol consumption and the characteristics of injuries among patients in rural Sub-Saharan Africa, specifically Moshi, Tanzania. Our study found that a large proportion (30%) of injured patients presenting to the ED were BAC positive. We found that men who were employed were more likely to be alcohol positive, and a significant association between alcohol use and injury risk was observed, mirroring other international research [4, 12, 28]. However, we found a relatively high prevalence of BAC-positive injuries (30%), compared with pooled international data (20%). Finally, we found an increased odds of injury due to alcohol use (OR 5.71; 95% CI, 3.84–8.50).

Overall, 30% of the participants were alcohol positive on arrival, validating prior work from the KCMC ED, which found that 28% of traumatic brain injuries were alcohol related according to history or physical exam. [15] International data have shown that 6–45% of ED injury patients have a positive alcohol test in the ED, with a pooled proportion of 20.4%. Only New Zealand and South Africa reported higher rates than our data (36, and 45%, respectively). [12] Our finding of alcohol-positive injuries in 30% of patients in Moshi, Tanzania is only slightly higher than other Sub-Saharan African countries. In Eldoret, Kenya, the proportion of BAC-positive patients was 23%. In Lusaka, Zambia, 26.7% of injury patients had a BAC above the legal limit of 80 mg/dL [29, 30]. Because most of our patients arrived at the hospital approximately 2.5 h after the injury, reflecting a substantial delay in care, we used any positive alcohol result rather than testing above the legal limit to be more conservative. However, 15.1% of our total population and 48.1% of our alcohol-positive population had BAC levels above the legal limit of 80 mg/dL. Compared with Tanzanian police data, which indicate that 1% of road traffic incidents are alcohol related, our data suggest that 29.1% of RTI patients were alcohol positive [31]. This discrepancy is likely due to limited testing resources and practices among the Tanzanian police, which do not routinely screen for alcohol use among motorists.

Concerningly, 23.7% (*n* = 37) of our alcohol-positive participants denied drinking alcohol around the time of the injury, and two participants reported they had not drank in the year prior to injury. While other studies have found discordance between objective and subjective testing for drugs, in individuals with a positive objective alcohol test, 0% denied alcohol use based on the AUDIT, showing complete concordance [32]. This finding suggests that there is reticence in reporting alcohol use to our practitioners, and potentially a stigma associated with alcohol use in the Tanzanian context. Similarly, this finding suggests that alcohol testing is necessary for diagnostic and intervention purposes in our setting. Given the relatively high proportion of abstainers within the country, self-disclosing alcohol use may be considered less desirable. Alcohol testing is a critical component of any surveillance system seeking to assess the proportion of drinkers or alcohol-related adverse outcomes.

Our study estimates an overall increased odds of injury of 5.7 due to alcohol, which is identical to the World Health Organization’s Collaborative Studies on Alcohol and Injuries pooled data analysis (OR 5.7, 95% CI: 4.5–7.3) for injury due to alcohol [9]. This finding validates a strong link between alcohol use and an increased odds of injury across countries and patient populations. Additionally, our data show a less clear dose-related pattern between the number of servings and an increased injury risk. For all injuries and for RTI specifically, we found an increase in the odds of injury with an incremental increase in the dosage of alcohol as seen in prior studies [11]. Our data are different from prior findings by Taylor et al. [11], as we did not find this pattern with intentional injuries. Taylor found that the highest per drink increase in odds of injury was for intentional injury using similar methods as our study. Our data likely differ from Taylor’s findings because of our sample size; the majority (*n* = 375) of our patients suffered RTIs, with only 88 suffering intentional injury; therefore, the alcohol dose subgroups are smaller in this category. Pooled or multicenter future studies would further delineate the strength of the dose-dependent OR for these injury subgroups.

### Strengths and limitations

While this study is a first of its kind in the region, the findings should be interpreted while considering a few important limitations. This study was limited to patients who suffered non-fatal injuries, those who sought care at the KCMC ED, and those who were able to obtain transportation to KCMC within 6 h of injury as required by our standard multi-national methodology [17]. The vast majority of patients (74.4%) who were not able to be enrolled in our study were excluded because they could not reach the hospital within 6 h of their injury. It is possible that those who were injured and intoxicated had more difficulty securing transportation to the hospital in the given timeframe, and this limitation could have falsely reduced the proportion of patients who were alcohol positive among our population. Comparing age and gender, prior studies have found that injury patients at KCMC have an average age of 34.6 years (SD 19.9) and 73.8% are males [14], which is approximately identical to our findings (mean age of 34.4 years (SD 13.3) and 76.4% male; these results suggest that our sample represents KCMC’s injury population despite the restrictive inclusion criteria for this study. While this is a representative sampling of patients who attended the KCMC ED within those time limits, a sampling bias may limit generalizability to the overall community because no prehospital care is available in Tanzania, and obtaining timely transport and care at this regional referral hospital is costly and difficult.

Our case-crossover survey method used self-report of alcohol use, which might have led to recall bias. Patients may not recall information 1 week prior [26], or even the day prior; however, they may recall amounts of alcohol on the day of injury. This differential recall may lead to an overestimation of the association between alcohol and injury. However, this methodology has been used successfully in multiple previous studies [9, 17]. Social desirability biases in Tanzania may lead to discongruous BAC and alcohol reporting; for instance, exaggerated responses regarding alcohol consumption may minimize legal implications or excuse socially unacceptable behavior [33]. Further research is warranted to understand these reporting practices in our setting. While our case-crossover design accounted for confounders within a patient, certain concurrent events or illnesses may impact both alcohol use and risk for injury. However, given the markedly elevated OR, and the validation with other studies, it is unlikely that these variations would substantially alter our key findings and implications.

### Conclusion

Of all the injury patients who presented to the KCMC ED for treatment of their injury, 30% tested positive for alcohol. Our data suggest that alcohol use is associated with a greater than 5-fold increase in the odds of injury, and a dose-dependent relationship between odds of injury and the amount of alcohol ingested was observed. Unfortunately, given the high burden of alcohol and injuries as demonstrated by our data, alcohol research, treatment, and prevention strategies are sorely lacking in Tanzania. Our empirical data can be used to grow the portfolio of alcohol research and prevention initiatives in the region.

## References

[1] Lim SS, Vos T, Flaxman AD, Danaei G, Shibuya K, Adair-Rohani H, AlMazroa MA, Amann M, Anderson HR, Andrews KG (2012). A comparative risk assessment of burden of disease and injury attributable to 67 risk factors and risk factor clusters in 21 regions, 1990: a systematic analysis for the global burden of disease study 2010. Lancet.

[2] World Health Organization, MoSA Unit: Global status report on alcohol and health, 2014. Geneva: World Health Organization; 2014.

[3] Rehm J, Mathers C, Popova S, Thavorncharoensap M, Teerawattananon Y, Patra J (2009). Global burden of disease and injury and economic cost attributable to alcohol use and alcohol-use disorders. Lancet.

[4] Rehm J, Room R, Monteiro M, Gmel G, Graham K, Rehn N, Sempos CT, Jernigan D (2003). Alcohol as a risk factor for global burden of disease. Eur Addict Res.

[5] Casswell S, Thamarangsi T (2009). Reducing harm from alcohol: call to action. Lancet.

[6] Organization WH (2005). Alcohol and interpersonal violence.

[7] Sorsdahl K, Stein DJ, Myers B (2012). Negative attribution towards people with substance use disorders in South Africa; variation across substances and by gender. BMC Psychiatry.

[8] Kaminer D, Grimsrud A, Myer L, Stein DJ, Williams DR (2008). Risk for post-traumatic stress disorder associated with different forms of interpersonal violence in South Africa. Soc Sci Med.

[9] Cherpitel CJ, Borges G, Giesbrecht N, Hungerford PM, Poznyak V, Room R, Stockwell T (2009). Alcohol and injuries: emergency department studies in an international perspective.

[10] Cherpitel CJ, Bond J, Ye Y, Borges G, Macdonald S, Giesbrecht N (2003). A cross-national meta-analysis of alcohol and injury: data from the emergency room collaborative alcohol analysis project (ERCAAP). Addiction.

[11] Taylor B, Irving HM, Kanteres F, Room R, Borges G, Cherpitel CJ, Bond J, Greenfield T, Rehm J (2010). The more you drink, the harder you fall: a systematic review and meta-analysis of how acute alcohol consumption and injury or collision risk increase together. Drug Alcohol Depend.

[12] Organization WH, Consumption ECoPRtA. WHO expert committee on problems related to alcohol consumption: second report: World Health Organization; 2007. 17970166

[13] Borges G, Fau CC, Orozco R, Fau OR, Bond J, Fau BJ, Ye Y, Fau YY, Macdonald S, Fau MS, Giesbrecht N, Fau GN, Stockwell T, Fau ST, Cremonte M, Fau CM, Moskalewicz J, Fau MJ, Swiatkiewicz G (2006). **Acute alcohol use and the risk of non-fatal injury in sixteen countries**. Addiction.

[14] Casey ER, Muro F, Thielman NM, Maya E, Ossmann EW, Hocker MB, Gerardo CJ (2012). Analysis of traumatic injuries presenting to a referral hospital emergency Department in Moshi, Tanzania. Int J Emerg Med.

[15] Staton CA, Msilanga D, Kiwango G, Vissoci JR, de Andrade L, Lester R, Hocker M, Gerardo CJ, Mvungi M (2017). A prospective registry evaluating the epidemiology and clinical care of traumatic brain injury patients presenting to a regional referral hospital in Moshi, Tanzania: challenges and the way forward. Int J Inj Control Saf Promot.

[16] Mitsunaga T, Larsen U (2008). Prevalence of and risk factors associated with alcohol abuse in Moshi, northern Tanzania. J Biosoc Sci.

[17] World Health Organization. WHO collaborative study on alcohol and injuries: final report. Geneva: World Health Organization; 2007.

[18] Borges G, Cherpitel C, Fau-Orozco R, Orozco R, Fau-Bond J, Bond J, Fau-Ye Y, Ye Y, Fau-Macdonald S, Macdonald S, Fau-Rehm J, Rehm J, Fau-Poznyak V, Poznyak V (2006). Multicentre study of acute alcohol use and non-fatal injuries: data from the WHO collaborative study on alcohol and injuries. Bull World Health Org.

[19] Cherpitel C, GR GN, Grant M, Osterberg R, Room R, Rootman I, Towle L (1989). A study of alcohol use and injuries among emergency room patients. Drinking and sasualities: accidents, poisoning and violence in an international perspective.

[20] Harris PA, Taylor R, Thielke R, Payne J, Gonzalez N, Conde JG (2009). Research electronic data capture (REDCap)—a metadata-driven methodology and workflow process for providing translational research informatics support. J Biomed Inf.

[21] Kiwango G, Msilanga D, Hocker M, Gerardo C, Lester R, Mvungi M, Ntabaye M, Lynch CA (2013). Epidemiology of traumatic brain injury patients at Kilimanjaro Christian medical centre, Moshi, Tanzania. Afr J Emerg Med.

[22] Van Buuren S, Groothuis-Oudshoorn K: MICE: multivariate imputation by chained equations in R. J Stat Softw 2011, 45(3):1-67.

[23] Maclure M (1991). The case-crossover design: a method for studying transient effects on the risk of acute events. Am J Epidemiol.

[24] Rothman KJ, Greenland S, Lash TL (2008). Modern Epidemiology.

[25] Ye (2013). Evaluating recall bias in a case-crossover design estimating risk of injury related to alcohol: data from six countries. Drug Alcohol Rev.

[26] Cherpitel CJ, Ye Y, Stockwell T, Vallance K, Chow C. Recall bias across 7 days in self-reported alcohol consumption prior to injury among emergency department patients. Drug Alcohol Rev. 2017; n/a-n/a. [Epub ahead of print] 10.1111/dar.12558 PMC5670029 28470876

[27] R, Team C (2014). R: a language and environment for statistical computing.

[28] Poznyak V, Peden M (2007). Alcohol and injury in emergency departments: summary of the report from the WHO collaborative study on alcohol and injuries.

[29] Patel NS. Traffic fatalities in Lusaka, Zambia. Med Sci Law. 1979;19(1):61–5. 10.1177/002580247901900110 759796

[30] Odero W (1998). Alcohol-related road traffic injuries in Eldoret, Kenya. East Afr Med J.

[31] Museru LMMC, Leshabari MT (2002). Road traffic accidents in Tanzania: a ten year epidemiological appraisal. East Central Afr J Surg.

[32] Sakai Lm Fau, Sakai LM, Esposito Tj Fau, Esposito TJ, Ton-That Hh Fau, Ton-That HH, Omi Ec Fau, Omi EC, Kovacs Ej Fau, Kovacs EJ, Schermer Cr Fau, Schermer CR: Comparison of objective screening and self-report for alcohol and drug use in traumatically injured patients. (0734-7324 (Print)). 10.1080/07347324.2012.718959 PMC4706078 26752806

[33] Davis CG, Thake J, Vilhena N (2010). Social desirability biases in self-reported alcohol consumption and harms. Addict Behav.

